# Diagnostic and prognostic value of stem cell factor plasma level in glioblastoma multiforme patients

**DOI:** 10.1002/cam4.4073

**Published:** 2021-07-11

**Authors:** Amirhosein Kefayat, Alireza Amouheidari, Fatemeh Ghahremani, Zahra Alirezaei

**Affiliations:** ^1^ Department of Oncology Isfahan University of Medical Sciences Isfahan Iran; ^2^ Department of Radiation Oncology Isfahan Milad Hospital Isfahan Iran; ^3^ Department of Medical Physics and Radiotherapy School of Paramedicine Arak University of Medical Sciences Arak Iran; ^4^ Department of Medical Physics and Radiotherapy Isfahan University of Medical Sciences Isfahan Iran

**Keywords:** biomarker, c‐Kit, diagnosis, glioblastoma multiforme, prognosis, stem cell factor

## Abstract

**Background:**

Investigation of novel blood‐circulating agents as potential biomarkers for glioblastoma multiforme (GBM) patients’ diagnosis and monitoring has gained lots of attention, due to limitations of imaging modalities and invasive tissue biopsy procedures. The present study aims to assess the diagnostic and prognostic values of preoperative stem cell factor (SCF) plasma level in GBM patients.

**Methods:**

Preoperative plasma samples from 58 GBM patients and 20 patients with nonglial tumors and 30 healthy controls were obtained. SCF levels were measured by employing the enzyme‐linked immunosorbent assay test and the values were compared between these three groups. Then, the association of SCF plasma level and tumor volume, progression‐free survival (PFS), and overall survival (OS) for the GBM patients were evaluated.

**Results:**

Mean preoperative SCF plasma level of the GBM patients (2.80 ± 1.52 ng/ml) was significantly higher (*p* < 0.0001) than the healthy controls (0.80 ± 0.24 ng/ml) and patients with nonglial tumor (1.41 ± 0.76 ng/ml). Receiver operating characteristic analysis revealed that the preoperative SCF plasma level could distinguish the GBM patients from healthy controls and patients with nonglial tumors with the area under curve values of 0.915 and 0.790, respectively. However, no significant association was observed between the GBM patients’ preoperative SCF plasma levels and tumors’ volume (Spearman Rho correlation coefficient, 0.1847; 95% CI, *p* = 0.1652). The GBM patients were divided into two subgroups based on mean preoperative SCF plasma levels (2.80 ng/ml). No significant difference was observed between the patients’ PFS (*p* = 0.3792) and OS (*p* = 0.1469) at these two subgroups.

**Conclusion:**

Taking together, the SCF plasma level can serve as a novel diagnostic blood‐circulating biomarker for patients with GBM. However, its plasma level is not correlated with GBM patients’ tumor volume, PFS, or OS.

## Graphical abstract

This study suggests that measurement of the SCF plasma level can aid in GBM patient's management for diagnostic purposes and as an efficient diagnostic plasma biomarker in patients with a new brain mass to differentiate between glioblastoma and nonglial tumors. However, this plasma biomarker does not exhibit prognostic value.

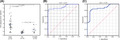

## 1 INTRODUCTION

Glioblastoma multiform (GBM) is the most aggressive and common type of primary brain tumors in human. GBM is often referred to as a grade IV astrocytoma.^1^ Its therapeutic approach consisted of surgical resection and postoperative radiotherapy with concurrent and/or adjuvant chemotherapy. However, GBM patients exhibit a median survival of 15 months and a mean 5‐year survival rate of <10%.^2^, ^3^

Diagnosis and monitoring of GBM patients typically rely on imaging techniques. However, imaging modalities are unable to differentiate between tumor progression and pseudoprogression which are some treatment‐related changes mimicking tumor progression. This can lead to misinterpretation of therapy response and delay clinical interventions. Therefore, the assessment of molecular markers at the tumor tissue is necessary for GBM diagnosis and predicting patient's prognosis and their response to treatment.^4^, ^5^, ^6^ However, neurosurgical procedures for obtaining tumor tissue are so invasive and with complications. Therefore, only patients in the good general condition, who their tumors are in noncritical parts of brain can undergo these procedures. Furthermore, tissue specimens from a single portion of GBM tumor cannot represent the whole tumor condition, due to GBM heterogenicity. Also, real‐time assessment of tumor tissue dynamics via multiple biopsies throughout treatment, often cannot be performed due to high invasiveness and risk for the patient. Therefore, identifying new tumor biomarkers at the bloodstream has gained lots of attention due to ease of access, low cost, and minimal invasiveness.^5^, ^7^, ^8^

Serum tumor biomarkers have undeniable roles in cancer diagnosis and treatment. These soluble molecules are secreted into the patient's circulation by malignant cells or their associated normal cells at the tumor microenvironment.^9^ An appropriate serum tumor biomarker should be able to (i) early detect tumor presence; (ii) predict response or resistance to the used therapeutic approach; (iii) monitor the patient after primary therapy; and (iv) predict the patient's prognosis.^10^, ^11^, ^12^ Currently, different serum tumor markers including carcinoembryonic antigen (CEA), cancer antigen 15–3 (CA15‐3), α‐fetoprotein (AFP), and prostate‐specific antigen (PSA) are used in clinical oncology practice.^13^, ^14^

Stem cell factor (SCF, also is known as steel factor and c‐Kit ligand) is a dimeric molecule that can bind and activate the c‐Kit receptor (also, referees to CD117). SCF plays key roles as a growth factor in promoting and regulating cells’ viability, proliferation, and differentiation in different biological processes in the human body.^15^ Moreover, the uncontrolled activity of the SCF/c‐Kit pathway can cause the formation of different human malignancies.^16^ The SCF/c‐Kit pathway is often overexpressed in different types of human tumors and can enhance tumorigenesis, proliferation, angiogenesis, stemness, and metastasis.^17^, ^18^, ^19^ Also, some studies have reported the relation of SCF/c‐Kit expression and tumor aggressiveness and patients’ poor prognosis.^16^, ^20^, ^21^, ^22^ In addition, activated SCF/c‐Kit pathway is related to tumor resistance to treatment.^23^, ^24^, ^25^ Sun et al. demonstrated that SCF expression by glioma cells can activate brain microvascular endothelial cells and consequently increase brain tumors angiogenesis in animal models.^19^ They reported that not only primary human gliomas express SCF per se (which was completely dependent to their grade), but also make normal neurons to express SCF in brain regions which has been invaded by glioma cells. Therefore, these regions usually exhibited high concentration of microvasculature according to Sun et al. observations. Moreover, SCF downregulation could significantly inhibit glioma tumors proliferation and growth progression in the tumor‐bearing animals which was deeply related to decrease of the tumor‐mediated angiogenesis. According to their study, overexpression of SCF was significantly associated with shorter survival time in GBM patients.

According to the best of our knowledge, different studies have reported the expression of SCF/c‐Kit pathway in GBM tumors.^26^, ^27^ However, the current knowledge about the association of SCF serum level and determinative parameters in the GBM patients’ clinical practice is limited. Therefore, the main aim of the present study is to evaluate the diagnostic sensitivity of the SCF plasma level for GBM and the correlation of this biomarker with the GBM patients’ tumor volume, prognosis, and overall survival to evaluate its significance as an independent blood‐circulating diagnostic and prognostic biomarker.

## 2 MATERIALS AND METHODS

### 2.1 Human ethics and informed consent

All procedures and methods were in accordance with the last version of the Helsinki Declaration.^28^ All patients were completely awared of the study and its purposes. The written informed consent was obtained from all patients and healthy controls to approve their agreement before involving in the study.

### 2.2 Study population

A total of 58 newly diagnosed GBM patients were included in this study. All the patients received the best approved therapeutic approach at the time of diagnosis which was consisted of surgery and radiotherapy with concomitant and adjuvant temozolomide. Multiple magnetic resonance imagines (MRI) were performed for patients’ before and after surgery. All tumors were examined histopathologically and classified according to the World Health Organization (WHO) classification. Besides, 20 patients with different types of nonglial brain tumors and 30 healthy controls with no recent history of head injury or symptoms or signs of an intracranial lesion were included in this study. The written informed consent was obtained from all patients and healthy controls before involving in the study.

### 2.3 Plasma samples collection

The blood samples of patients with GBM and nonglial tumors were collected exactly before surgery. Also, the healthy volunteers’ samples were taken in the absence of a concurrent inflammatory illness at the time of the appointment. Blood was drawn into tubes that contained a one‐tenth volume of 0.1 M sodium citrate as an anticoagulant to separate plasma. Then, the samples were centrifuged at 3000 × g for 10 min. The supernatant fluid was collected and aliquoted into 2 ml cryotubes and stored in liquid nitrogen for further experiments.

### 2.4 DNA extraction and molecular evaluation

The QIAamp DNA Mini Kit (Qiagen) was employed to extract DNA from frozen tumor specimens. Subsequently, a Nano‐Drop ND‐1000 spectrophotometer was used to measure the DNA concentration and quality. The isocitrate dehydrogenase 1 and 2 genes (IDH1/2) mutation status (DNA pyro‐sequencing) and the MGMT (O6–methyl guanine methyl transferase gene) promoter methylation status (DNA pyro‐sequencing) were assessed based on previously published studies.^29^, ^30^

### 2.5 Enzyme‐linked immunosorbent assay (ELISA)

All the stored plasma samples were analyzed for SCF at once. All the stored samples were equilibrated to the room temperature and diluted 1:2 into 1X Diluent M. Then, they were assessed, blind to the clinical data, by employing the human SCF ELISA kit (ab108901, Abcam). All samples were tested in duplicate according to the manufacturer's instructions. The absorbance was read on a microplate reader (Bio‐Rad 680) at a wavelength of 450 nm. To generate the standard curve, the prepared standard concentrations (0, 0.156, 0.625, 2.500, 10.00, and 40.00 ng/ml) were put on the x‐axis and the corresponding mean at 450 nm absorbance on the y‐axis. According to the manufacturer's instructions, the minimal detectable dose of SCF for this kit is 0.09 ng/ml and its standard range of detectable dose is from 0.156 ng/ml to 40 ng/ml. This kit is specific for human SCF and percentage of cross reactivity with other species including beagle, bovine, mouse, rat, and swine is zero and just for monkey is <10. Intra‐assay and inter‐assay precisions (CV%) of the kit are 4.5 and 7.2, respectively. Its average recovery is 96% in a range of 86%–107%. For the linearity of the plasma dilution (no dilution, 1:2, 1:4), the average % expected values are between 98% and 102%.

### 2.6 Tumor volume estimation

Preoperative post‐contrast T1‐weighted MRIs of the GBM patients were analyzed for tumor volume estimation. For this purpose, the modified ellipsoid equation (1) was used according to previous studies.^31^ In this equation, *A*, *B*, and *C* represent the largest perpendicular diameters of the enhancing lesion.

(1) $$Tumor^{\prime }svolume=\frac{A\times B\times C}{2}$$

### 2.7 Statistical analysis

All statistical analyses were performed by employing JMP 14.0 software (SAS Institute). The statistical differences between the preoperative SCF plasma levels of the GBM patients, patients with nonglial tumors, and healthy controls were assessed using a one‐way analysis of variance (ANOVA). The logistic model was employed to evaluate the discriminating diagnostic value of the SCF plasma level between the GBM patients, patients with nonglial tumors, and healthy controls. The area under the curve (AUC) of the receiver operator characteristic (ROC) was analyzed. The association between the SCF plasma level and tumor volume in GBM patients was assessed by the Spearman correlation coefficient. Overall survival (OS) was calculated from the date of surgery until death or last follow‐up. Progression‐free survival (PFS) was defined as the time from surgery to disease progression or death. Kaplan–Meier survival analyses using the log‐rank test were performed to investigate the differences between PFS and OS of the GBM patients with SCF plasma levels above the mean and those below the mean.

## 3 RESULTS

### 3.1 Patients’ characteristics

Among 58 GBM patients who were included in this study, 30 patients were male (52%) and 28 were female (48%). The patients’ median age was 60 years and their ages ranged from 30 to 81 years (Table 1). The median estimated tumor volume of the GBM patients according to the preoperative MRIs was 23.8 cm^3^ (range, 4.5–112.5 cm^3^). The preoperative Karnofsky Performance Scale (KPS) score for about 57% of the enrolled GBM was evaluated to be ≥80. Based on surgery reports and post‐surgery MRIs, 18 patients (31%) underwent complete resection surgery. This number for partial resection and biopsy were 17 (29%) and 23 patients (40%), respectively. Among the patients, three (5%) persons were diagnosed with secondary glioblastoma. The best approved therapeutic approach at the time of diagnosis was used for the patients. For all of the involved GBM patients in this study, the status of IDH1/2 mutation and MGMT promoter methylation were analyzed. Mutated IDH1/2 was observed in 10 cases (17%) and 22 cases (38%) were identified with methylated MGMT promoter. The GBM patients’ median PFS and OS were 9.9 months (95% confidence interval (95% CI), 7.8–12.6 months) and 17.1 months (95% CI, 11.8–25.1 months), respectively. Of 20 patients with nonglial intracranial tumors, 11 were male (55%) and 9 were female (45%). Their median age was 58.5 years (range, 34–82 years). Histopathological evaluations exhibited that the patients with nonglial tumors consisted of 10 meningioma patients (50%), 7 brain metastasis patients (35%), 2 patients (10%) with the diagnosis of primary central nervous system lymphoma, and 1 patient (5%) with the diagnosis of medulloblastoma. Also, 30 healthy controls were included in this study as controls. Of these healthy controls, 17  persons were male (57%) and 13 were female (43%). Their median age was 56 years (range, 25–81 years). All the GBM patients, patients with nonglial tumors, and healthy controls’ characteristics are summarized in Table 1.

**TABLE 1:** Study population's characteristics

Parameters	Healthy controls (*n *= 30)	Nonglial tumors (*n *= 20)	GBM (*n *= 58)
Age, y			
Median (range)	56 (25–81)	58.5 (34–82)	60 (30–81)
Gender, *n* (%)			
Male	17 (43)	11 (55)	30 (52)
Female	13 (57)	9 (45)	28 (48)
Preoperative KPS score, *n* (%)			
<80	—	—	25 (43)
≥80	—	—	33 (57)
Preoperative tumor volume, *n* (cm^3^)			
Median (range)	—	—	23.8 (4.5–112.5)
Extend of surgery, *n* (%)			
Biopsy	—	—	23 (40)
Partial resection	—	—	17 (29)
Total resection	—	—	18 (31)
IDH1/2 mutation status, *n* (%)			
Mutated	—	—	10 (17)
Wild type	—	—	48 (83)
MGMT promoter methylation status, *n* (%)			
Methylated	—	—	22 (38)
Not methylated	—	—	36 (62)
PFS, mo			
Median (range, 95% CI)	—	—	9.9 (7.8–12.6)
OS, mo			
Median (range, 95% CI)	—	—	17.1 (11.8–25.1)

Abbreviations: 95% CI, 95% confidence interval; GBM, glioblastoma multiforme; IDH1/2, isocitrate dehydrogenase 1 and 2 genes; KPS, Karnofsky Performance Scale; MGMT, O6–methyl guanine methyl transferase gene; mo, month; *n*, number; OS, overall survival; PFS, progression‐free survivaly, years.

### 3.2 SCF plasma level in the patients with GBM, patients with nonglial tumors, and healthy controls

**FIGURE 1:**
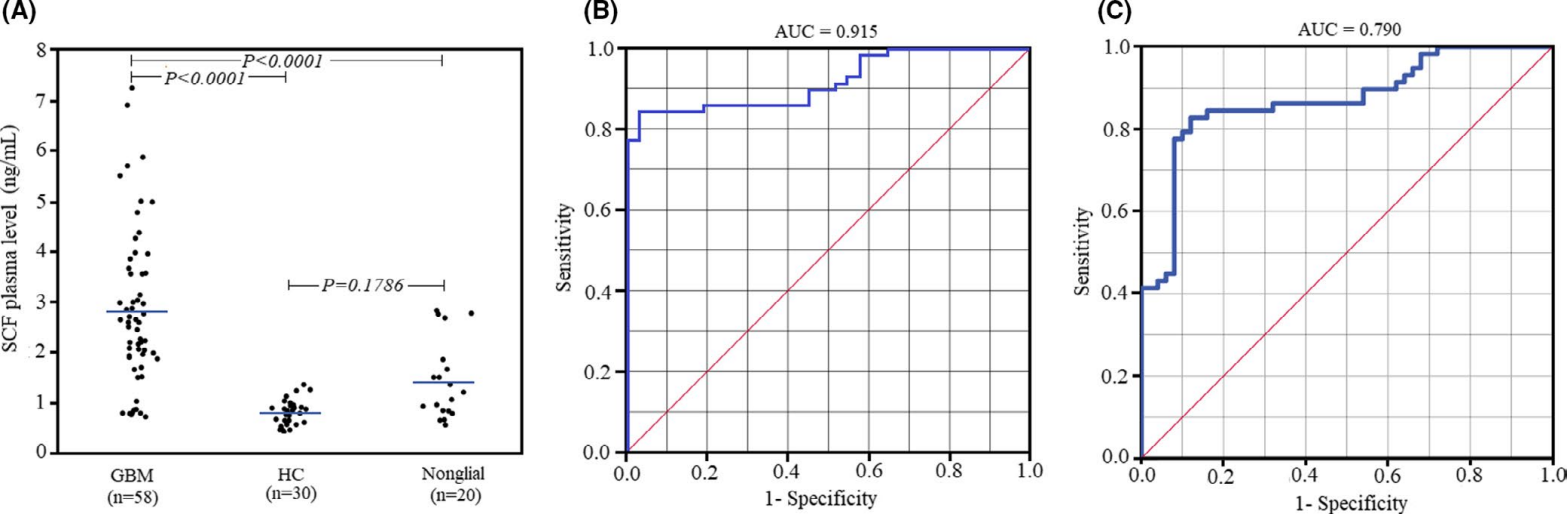
(A) The dot plot of preoperative SCF plasma levels of the GBM patients (*n* = 58), patients with nonglial tumors (*n *= 20), and healthy controls (*n *= 30). (B) Receiver operating characteristics (ROC) plot to illustrate the predictive value of the SCF plasma level for discrimination between the GBM and healthy controls. (C) ROC curve to illustrate the diagnostic ability of the SCF plasma level for discrimination between the GBM patients and patients with nonglial tumors

SCF level was assessed in the preoperative plasma of the GBM patients and compared with the patients with nonglial tumors and healthy controls. As Figure 1A illustrates, the GBM patients exhibited a significantly (*p* < 0.0001) higher mean SCF plasma level (2.80 ± 1.52 ng/ml) than the healthy controls (0.80 ± 0.24 ng/ml). A statistically significant difference (*p* < 0.0001) was observed between the mean preoperative SCF plasma level of the patients with GBM (2.80 ± 1.52 ng/ml) and nonglial tumors (1.41 ± 0.76 ng/ml). Also, receiver operating characteristics (ROC) analysis was used to evaluate the predictive value of the SCF plasma level for discrimination between the GBM patients, healthy controls (Figure 1B), and patients with nonglial tumors (Figure 1C). ROC analysis revealed that the preoperative SCF plasma level could distinguish the GBM patients from healthy control and patients with nonglial tumors with the area under curve (AUC) values of 0.915 and 0.790, respectively. Therefore, the SCF plasma level can be identified as an efficient diagnostic plasma biomarker in patients with a newly diagnosed brain mass to differentiate between glioblastoma and nonglial tumors.

### 3.3 Association of preoperative SCF plasma level and GBM tumor's volume, IDH1/2 mutation status, and MGMT promoter methylation status

**FIGURE 2:**
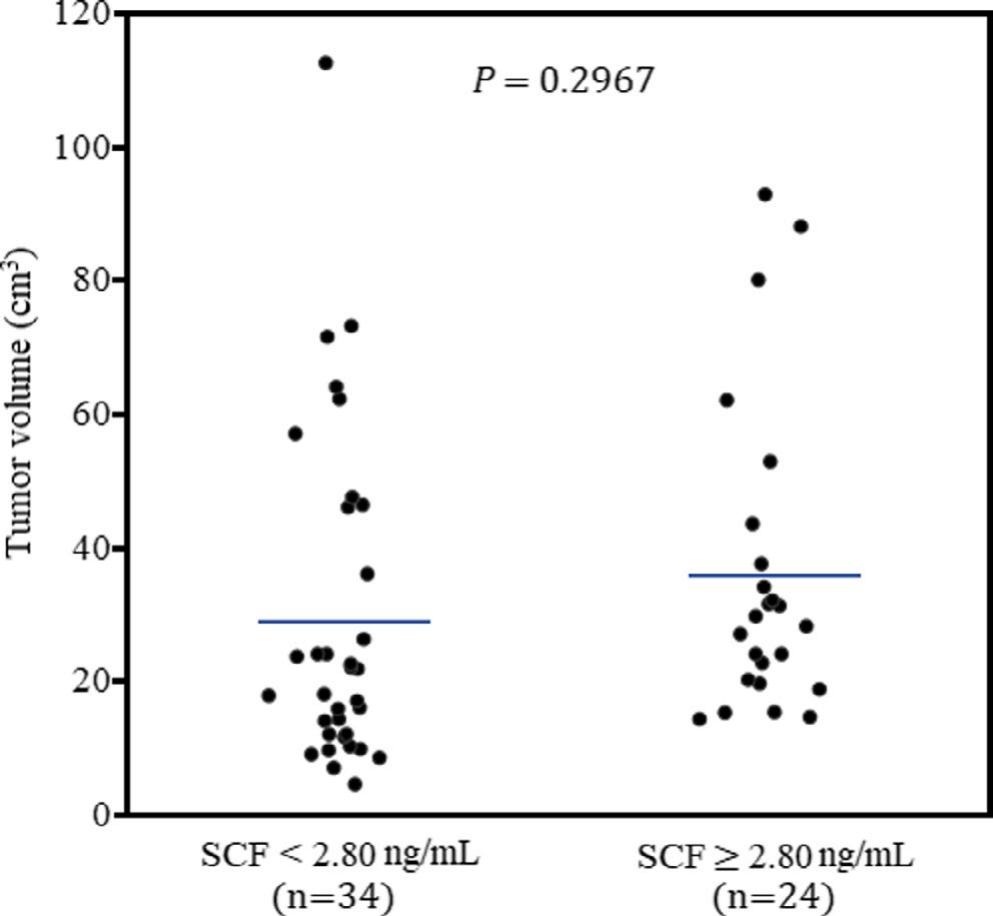
Association of preoperative SCF plasma level and tumor volume in the GBM patients

The correlation between preoperative SCF plasma level and GBM patients’ tumor volume was assessed by the Spearman Rho correlation coefficient. No significant association was found between GBM patients’ preoperative SCF plasma levels and tumor volume (Spearman Rho correlation coefficient, 0.1847; 95% CI, *p* = 0.1652). According to previous studies, the mean value of the selected plasma biomarker was accounted as the threshold divided the GBM patients into two groups.^31^ Therefore, the GBM patients were divided into two groups including SCF ≥2.80 ng/ml and SCF <2.80 ng/ml. The patients’ characteristics of these two subgroups are mentioned in Table 2. As Figure 2 illustrates, the patients with SCF plasma levels above the mean value did not exhibit significantly (*p* = 0.2967) higher tumor volumes (n:24, mean: 35.78 ± 23.03 cm^3^) in comparison with the patients with below the threshold levels of plasma SCF (n:34, 29.04 ± 23.03 cm^3^). Moreover, no difference was observed at KPS score (*p* = 0.7914), IDH1/2 mutation status (*p* = 0.5624), and MGMT promoter mutation status (*p* = 0.5929) of these two subgroups.

**TABLE 2:** Patients characteristics at SCF ≥2.80 ng/ml and SCF <2.80 ng/ml subgroups of GBM patients

Parameters	SCF ≥2.80 (*n*=24)	SCF <2.80 (*n*=34)
Age, y		
Median (range)	61 (37–81)	60 (30–79)
Gender, *n* (%)		
Male	12 (50)	18 (53)
Female	12 (50)	16 (47)
Preoperative KPS score, *n* (%)		
<80	11 (46)	14 (41)
≥80	13 (54)	20 (59)
Preoperative tumor volume, *n* (cm^3^)		
Median (range)	28.9 (4.5–112.5)	19.9 (14.2–92.8)
Extend of surgery, *n* (%)		
Biopsy	11 (46)	12 (35)
Partial resection	5 (21)	12 (35)
Total resection	8 (33)	10 (30)
IDH1/2 mutation status		
Mutated	4 (17)	6 (18)
Wild type	20 (83)	28 (82)
MGMT promoter methylation status		
Methylated	8 (33)	14 (41)
Not methylated	16 (67)	20 (59)
PFS, mo		
Median (range, 95% CI)	9.6 (7.8–12.6)	10.2 (7.9–12.4)
OS, mo		
Median (range, 95% CI)	17.1 (12.5–24.6)	17.5 (11.8–25.1)

Abbreviations: 95% CI, 95% confidence interval; GBM, glioblastoma multiforme; IDH1/2, isocitrate dehydrogenase 1 and 2 genes; MGMT, O6–methyl guanine methyl transferase gene; mo, month; *n*, number; OS, overall survival; PFS, progression‐free survival; y, years.

### 3.4 Association of preoperative SCF plasma level and GBM patient's PFS and OS

**FIGURE 3:**
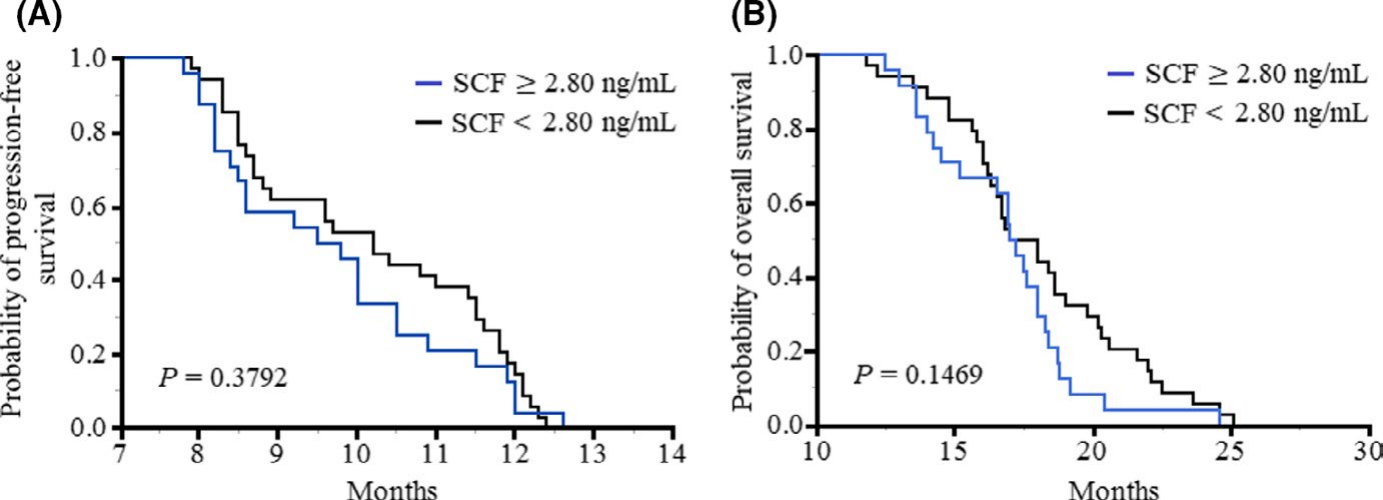
Kaplan–Meier (A) progression‐free survival and (B) overall survival curves of the GBM patients are shown according to the preoperative SCF plasma level. The mean value of the preoperative SCF plasma level was utilized as a cutoff point to divide the GBM patients into two groups including SCF ≥2.80 ng/ml and SCF <2.80 ng/ml

The mean value of the preoperative SCF plasma level was utilized as a cutoff point to divide the GBM patients into two groups including SCF ≥2.80 ng/ml and SCF <2.80 ng/ml. As Figure 3A illustrates, the median PFS was 10.2 months (95% CI, 7.9–12.4 months) for patients with levels below the threshold and 9.6 months (95% CI, 7.8–12.6 months) for the SCF ≥2.80 ng/ml group (*p* = 0.3792). As Figure 3B shows, the median OS of the GBM patients at the SCF <2.80 ng/ml and SCF ≥2.80 ng/ml groups was 17.5 months (95% CI, 11.8–25.1 months) and 17.1 months (95% CI, 12.5–24.6 months), respectively. Therefore, no significant difference was observed between the two groups of GBM patients for PFS (*p* = 0.3792) and OS (*p* = 0.1469) parameters.

## 4 DISCUSSION

Previous studies have investigated the diagnostic and prognostic value of SCF serum levels for different types of cancers. Mroczko et al. identified that SCF serum level can be an appropriate biomarker for non‐small cell lung carcinoma diagnosis and patients’ monitoring. They reported that its diagnostic sensitivity is related to the stage of the disease.^32^ Also, the SCF level was reported to be significantly higher in the colorectal cancer patients’ serum in comparison with healthy controls. Moreover, SCF serum level exhibited more diagnostic sensitivity than the current colorectal tumor markers including CEA and CA 19–9.^33^

Identification of alternative blood biomarkers with diagnostic and prognostic value can be extremely helpful in the GBM patients’ management, due to limitations of imaging modalities and invasive tissue biopsy procedures.^7^ Xu et al. quantified a list of different proteins in plasma and serum specimens from GBM patients and healthy subjects. Five of the proteins, which SCF was one of them, were identified as high‐efficient diagnostic biomarkers for patients with malignant glioma.^34^ Also, Sun et al. reported that overexpression of SCF in GBM tumors has significant correlation with the patients’ shorter survival time. They attributed these observations to pro‐angiogenetic effect of SCF.^19^

In the current study, we put one step beyond and investigated the diagnostic and prognostic significance of preoperative SCF plasma levels in GBM patients. According to our observations, the preoperative SCF plasma level was significantly higher in GBM patients in comparison with healthy and nonglial tumor‐bearing patients and exhibited high diagnostic sensitivity. However, the SCF level was not associated with the GBM patients’ tumor volume, PFS, and OS. Therefore, the preoperative SCF plasma level can help in GBM patient's management as a diagnostic biomarker and for differentiate between glioblastoma and nonglial tumors in a newly diagnosed brain mass. However, this plasma biomarker does not exhibit prognostic significance. Many other plasma proteins were reported as efficient diagnostic and prognostic biomarkers for GBM patients. Perez‐Larraya et al. investigated the diagnostic and prognostic significance of preoperative insulin‐like growth factor‐binding protein 2 (IGFBP‐2), chitinase‐3‐like protein 1 (YKL‐40), and glial fibrillary acidic protein (GFAP) plasma levels in GBM patients. A combined profile of the mentioned biomarkers exhibited high efficacy as a diagnostic tool for GBM suspicious brain lesions. Besides, IGFBP 2 level could play the role of an independent prognostic factor in GBM patients.^31^

Moreover, the correlation of SCF plasma level with other diseases' progression was previously reported. Makowska et al. demonstrated that SCF serum level can reflect disease severity in asthma patients.^35^ Kanbe et al. published some observations to demonstrate the association of SCF serum level with atopic dermatitis activity and severity.^36^ Mean SCF serum concentration was fivefold higher in chronic renal failure patients in comparsion with healthy controls. Also, it was significantly correlated with important clinical parameters of these patients including blood urea nitrogen, creatinine, and hemoglobin.^37^ Besides, different studies reported different mean normal values for SCF plasma levels according to their healthy controls’ samples analysis. Wodnar‐Filipowicz et al. reported 3.3 ± 1.0 ng/ml as the mean SCF plasma level in 257 healthy subjects (*n* = 257).^38^ Zhong et al. investigated the correlation between blood pressure and the SCF/c‐Kit plasma level. The mean value of SCF plasma level in the normal controls was 0.76 ± 0.04 ng/ml (*n* = 36).^39^ Kojima et al. reported 1.16 ± 0.31 ng/ml as the mean value of healthy subjects’ SCF plasma concentration (*n* = 20). The main aim of their study was comparing the SCF plasma level between healthy controls and patients with aplastic anemia.^40^ According to our measurements, the mean SCF plasma level in the healthy controls was 0.80 ± 0.24 ng/ml. Taking together, many further comprehensive studies are needed to solve these inconsistencies.

### 4.1 Conclusion

According to our observations, the preoperative SCF plasma level was founded to be significantly higher in GBM patients in comparison with healthy controls and patients with nonglial tumors. However, the level of this blood‐circulating protein was not associated with the GBM patients’ tumor volume, PFS, and OS. These results suggest that the measurement of the SCF plasma level can aid in GBM patient's management for diagnostic purposes and as an efficient diagnostic plasma biomarker in patients with a new brain mass to differentiate between glioblastoma and nonglial tumors. However, this plasma biomarker does not exhibit prognostic value. So, further studies are needed to evaluate this blood‐circulating biomarker efficacy at predicting the GBM patients’ prognosis. Maybe, a vast comprehensive study with a high number of involved participants for estimating the baseline SCF plasma level should be performed at first.

## CONFLICT OF INTERESTS

The authors declare no conflict of interest.

## HUMAN ETHICS AND INFORMED CONSENT

This study was conducted after approval of the ethics committee of Arak University of Medical Sciences (IR.ARAKMU.REC.1398.174).

## Data Availability

All data are available upon request.
